# Development and immunological assessment of VLP-based immunogens exposing the membrane-proximal region of the HIV-1 gp41 protein

**DOI:** 10.1186/s12929-014-0079-x

**Published:** 2014-08-27

**Authors:** Thomas D Benen, Paul Tonks, Alexander Kliche, Ruth Kapzan, Jonathan L Heeney, Ralf Wagner

**Affiliations:** Molecular Microbiology and Gene Therapy Unit, Institute of Medical Microbiology and Hygiene, University of Regensburg, Franz-Josef-Strauss-Allee 11, 93053 Regensburg, Germany; Department of Veterinary Medicine, University of Cambridge, Cambridge, UK; Current address: Malvern Instruments GmbH, Herrenberg, Germany; Current address: GeneArt AG / LifeTechnologies Inc, Regensburg, Germany

**Keywords:** HIV-1, gp41, VLPs, Vaccine, Immunization study, Neutralizing antibodies

## Abstract

**Background:**

The membrane-proximal external region (MPER) of HIV-1 gp41 is particularly conserved and target for the potent broadly neutralizing monoclonal antibodies (bnMAbs) 2F5, 4E10 and 10E8. Epitope focusing and stabilization present promising strategies to enhance the quality of immune responses to specific epitopes.

**Results:**

The aim of this work was to design and evaluate novel immunogens based on the gp41 MPER with the potential to elicit cross-clade neutralizing antibodies. For that purpose, gp41 was truncated N-terminally in order to dispose immunodominant, non-neutralizing sites and enhance the exposure of conserved regions. To stabilize a trimeric conformation, heterologous GCN4 and HA2 zipper domains were fused based on an in silico “best-fit” model to the protein’s amino terminus. Cell surface exposure of resulting proteins and their selective binding to bnMAbs 2F5 and 4E10 could be shown by cytometric analyses. Incorporation into VLPs and preservation of antigenic structures were verified by electron microscopy, and the oligomeric state was successfully stabilized by zipper domains. These gp41 immunogens were evaluated for antigenicity in an immunization study in rabbits primed with homologous DNA expression plasmids and boosted with virus-like particle (VLP) proteins. Low titers of anti-MPER antibodies were measured by IgG ELISA, and low neutralizing activity could be detected against a clade C and B viral isolate in sera.

**Conclusions:**

Thus, although neutralizing titers were very moderate, induction of cross-clade neutralizing antibodies seems possible following immunization with MPER-focusing immunogens. However, further refinement of MPER presentation and immunogenicity is clearly needed to induce substantial neutralization responses to these epitopes.

**Electronic supplementary material:**

The online version of this article (doi:10.1186/s12929-014-0079-x) contains supplementary material, which is available to authorized users.

## Background

The development of a preventive vaccine against human immunodeficiency virus type 1 (HIV-1) has been pursued by many research groups around the globe using a variety of approaches [1]. Broadly neutralizing antibodies (bnAbs) are thought to be a critical component of an effective immune response. Eliciting such antibodies by vaccination has proven to be difficult due to the substantial genetic variation of HIV-1 and its ability to evade host immune defenses by different mechanisms [2]. Although bnAbs alone may not be able to fully control HIV-1 replication once the infection has been established, they can protect at low titers if they are present in immunologically relevant sites, as has been shown in several passive immunization experiments in non-human primate studies [3].

Besides the surface protein gp120, the transmembrane protein gp41 is target for bnAbs during infection [4], rendering this protein a worthwhile target for vaccine design. According to the currently accepted model for HIV-1 fusion [5], following engagement of host cellular receptors, the viral envelope trimer undergoes conformational changes and inserts the amino-terminal fusion peptide (FP) of gp41 into the host cell membrane. The fusion mechanism involves two helical regions of gp41, the N-terminal heptad repeat (NHR) and C-terminal heptad repeat (CHR), which are associated to form a pre-hairpin intermediate. In this conformation, the gp41 molecules are extended forming a trimer of CHR helices and a trimer of NHR helices that bridge both the viral and cellular membranes. It subsequently collapses into a trimer-of-hairpins structure that pulls both membranes into tight apposition and induces membrane fusion. This structure consists of the six-helix bundle, in which three CHR peptides pack in an antiparallel manner against a central three-stranded coiled-coil formed by the NHR regions [6].

Three of the identified monoclonal Abs against gp41, denoted 2F5, 4E10 and 10E8, bind to adjacent epitopes located in the membrane-proximal external region (MPER) [7]. The MPER is indispensable for membrane fusion and viral entry, and is highly conserved among HIV-1 groups. It is accessible for IgGs already in the native envelope conformation, but thought to have higher accessibility during the fusion process [8]. Some residues may be buried within the membrane in pre-fusion and intermediate conformations [9]. Although 2F5 and 4E10 bind to linear core epitopes in the MPER, a correct three-dimensional display of their epitopes and proximal residues in the ectodomain might enhance the recognition [10]. Additionally to binding to the gp41 protein, both 2F5 and 4E10 can interact with membranes through hydrophobic residues in their heavy chain CDR3 regions. A model has been suggested, in which 4E10 first binds to the membrane, then recognizes its protein epitope and locks conserved residues by an induced-fit mechanism [9]. Removal of the membrane component decreases binding and neutralization of these special antibodies [11]. Taken together, these results suggest displaying the MPER in a native three-dimensional context, embedded into a membrane, for optimal mimicry of the natural epitopes in an immunogen.

Unfortunately, the MPER is weakly immunogenic, compared to the loop region of gp41. Past immunization studies demonstrated that MPER-containing proteins, fusion intermediate-mimicking proteins expressed on HIV-1 VLPs and chimeric proteins all failed to elicit MPER-specific neutralizing antibodies [12,13]. The antibody response was directed towards immunodominant, but non-neutralizing epitopes in these studies. Others were able to elicit high titers of MPER-targeting Abs by a soluble 6-helix bundle immunogen, however, neutralizing activity could only be detected after purification of IgG and not in unfractionated sera [14]. Thus, the problem of eliciting high titers of anti-MPER Abs with broadly neutralizing activity remains unsolved. A reduction of gp41 to its neutralizing epitopes, yet expanding those sites with additional residues responsible for membrane embedment and formation of the full epitopes, seems to be a promising strategy.

The objective of this work was to design and evaluate novel immunogens based on HIV-1 gp41 MPER which combine the above mentioned criteria. For this purpose, variants of truncated gp41 proteins consisting of CHR, MPER and TM regions were designed and characterized. Heterologous peptide sequences were fused for intracellular trafficking and efficient trimerization. The immunogens were designed to strongly expose the MPER with binding sites to known bnMAbs in a natural conformation and membrane environment. Plasmid DNA and pseudotyped virus-like particles (VLPs) were chosen as immunization vehicles in a rabbit model. After a DNA prime and VLP boost regimen, the presence of anti-MPER Abs and neutralizing activity in unfractionated sera was analyzed.

## Methods

### Software-assisted design of gp41 derivatives

An N- and C-terminally truncated gp41 (“gp41CTM”) was designed according to Lenz et al. [15]. Wild-type sequences for synthesis of viral gene fragments were obtained from GenBank and optimized for human codon usage (Life Technologies). Molecular clones of viral isolates HIV-1 89.6 (subtype B, accession number U39362), HIV-1 96ZM651 (subtype C, AF286224) and SIVmac239 (M33262) served as templates with aa 629–724 of 89.6 and 96ZM651 (HXB2 reference numbering) and aa 639–740 of SIVmac239 (SIVmac239 numbering). Membrane incorporation and orientation of all constructs was checked by the use of the transmembrane prediction TMHMM Server v. 2.0 [16]. A human tissue plasminogen activator (TPA) leader sequence was fused N-terminally. Correct cleavage of the leader sequence was predicted by SignalP 3.0 software [17]. A Kozak consensus sequence (GCCGCCACC) was inserted 5’ of the start codon for enhanced transcription. The sequence for an HA-tag from *Influenza* virus Hemagglutinin HA1 protein (YPYDVPDYA) was codon-optimized and inserted C-terminally by primer extension PCR for recognition of recombinant proteins (primers 3E8 & 2H4/2H5/2H6, see Additional file 1: Table S1 for primer sequences). The topology of all gene variants was checked with Phobius Prediction Server [18].

### Trimer-stabilized constructs and molecular visualization

Two zipper domains were inserted between the TPA leader and gp41-derived sequences by fusion PCR. One motif was derived from the HA2 protein (amino acids 372–417, primers 3H4 & 3H5/3H6/3H7) of human *Influenza* virus strain H3 followed by a linker composed of amino acids Gly-Ser-Thr [13]. A second domain was derived from the GCN4 protein of *Saccharomyces cerevisiae* [19] (primers 3B6 & 3B7/3B8/3B9). Sequences for GCN4 and H3 zippers were derived from Genbank (IDs: CY002064, BK006939) and codon-optimized. To confirm the correct gene fusion position of zippers to gp41 fragments, molecular models were built using Modeller and evaluated by calculating the DOPE score (Discrete Optimized Protein Energy [20]. Resulting atomic models were visualized using PyMOL Molecular Graphics System.

### SDS-PAGE, Western Blots and Slot Blots

SDS-PAGE was done with acrylamide concentrations of 12.5%. Western Blot transfers of proteins from SDS gels were done in a semi-dry system (Serva, Heidelberg, Germany) according to manufacturer’s instructions. For slot blots, protein solutions were loaded onto a Bio-Dot SF (Biorad) apparatus including 5 layers of pre-wetted Whatman Chromatography paper (Whatman International Ltd, Maidstone, UK). For both methods, nitrocellulose membranes with a pore size of 0.2 μm were applied (Millipore, Bedford, USA) and blocked in TBS (150 mM NaCl, 50 mM Tris/HCl, pH 7.4) containing 5% skim milk powder over night at 4°C. For antibody staining, blots were incubated for 1 h in TTBS (TBS + 0.3% (v/v) Tween-20) with primary antibody, washed four times for 15 min in TTBS, incubated for 1 h in TTBS with secondary antibody, washed again four times for 15 min in TTBS, and subjected to either ECL (2.5 mM luminol, 0.4 mM coumaric acid, 0.1 M TrisHCl pH 8.5 plus equal volume of 0.018% H_2_O_2_, 0.1 M TrisHCl pH 8.5) or alkaline phosphatase (AP) staining solution (5 mM TrisHCl pH 9.5, 5 mM NaCl, 2.5 mM MgCl_2_ plus 1/50 volume of NBT/BCIP stock (Roche)). AP reactions were stopped with excess of dH_2_O. ECL reactions were measured in a ChemiluxPro device (Intas, Göttingen, Germany). Intensity of bands was quantified with the aid of Gel-Pro Analyzer software (Media Cybernetics, Bethesda, USA). Human monoclonal antibodies (MAbs) 2F5 and 4E10 (diluted to 5 μg/ml each) served for detection of gp41 derivatives, the HA-tag was recognized by rat MAb 3F10 (“anti-HA High Affinity”, 0.1 μg/ml, Roche). Pr55/p24 was detected with mouse MAb M13/5 (cell culture supernatant, 1:500, [21]). Polyclonal HRP- or AP-coupled anti-human-IgG, anti-rat-IgG, and anti-mouse-IgG antibodies (all 1:2,000, all from Dako, Glostrup, Denmark) served as secondary antibodies.

### ELISA

The amount of Pr55 or p24 protein in lysates was quantified with the aid of an Enzyme-linked Immunosorbent Assay (ELISA) using MAb M01 (1:1000, Polymun, Vienna, Austria) as coating antibody [22]. An ELISA with gp41-derived peptides was used to quantify anti-gp41 immunoglobulins in animal sera by the end-point dilution method in duplicates [23]. Peptides spanning the MPER (EQNEKDLLALDSWNNLWNWFDITKWLWYIK) and CHR regions (MQWDREISNYTNTIYRLLEDSQSQQEQNEK, both from Pepscan Presto BV, Lelystad, Netherlands) were used for coating at 100 ng/ml on Nunc Maxisorp plates (Thermo Fisher Scientific, Waltham, USA). HRP-coupled polyclonal anti-rabbit-IgG and anti-human-IgG served as secondary antibodies (1:4,000, Dako). Washing was done with the aid of a high-throughput microplate washing device (MAP-C2 workstation, Titertek Instruments Inc., Huntsville, USA).

### Transient transfection

HEK 293 T (ATCC Microbiology Collections) were grown in Gibco Dulbecco’s Modified Eagle Medium (DMEM) supplemented with 10% fetal calf serum, 100 U/ml penicillin and 0.1 mg/ml streptomycin (Invitrogen). FreeStyle 293 F cells (Invitrogen) were grown in Gibco FreeStyle 293 expression medium (Invitrogen) supplemented with 50 U/ml penicillin and 0.05 mg/ml streptomycin at 125 rpm and handled according to the manufacturer’s instructions. HEK293T cells were seeded in 6-well plates (4x10^5^ cells in 2 ml medium). 24 h after seeding, medium was replaced by 1 ml of DMEM without supplements. Cells were transfected with the respective expression vector(s) according to the poly-ethylenimine (PEI) transfection method using a total of 2 μg DNA and 8 μl of a 1 mg/ml PEI in H_2_O solution ad 50 μl DMEM without supplements. Components were mixed, left standing for 10 min at room temperature and added drop-wise to the cell suspension. 4 h later, transfection medium was replaced with 2 ml of supplemented growth medium. For smaller (96-well) or larger (10 cm/15 cm Petri dishes) assays, the number of cells, the amount of DNA and PEI, and the volume of medium was down- or up-scaled, based on the vessel surface area. 293 F cells were adjusted to 1 million cells/ml in fresh FreeStyle medium and 30 ml of cell suspension were transferred to 125 ml flasks. 37.5 μg of DNA and 150 μl of PEI ad 1.8 ml DMEM without supplements were mixed for transfection of one flask. 4 h later, transfection medium was replaced with 30 ml of FreeStyle medium supplemented with 100 U/ml penicillin and 0.1 mg/ml streptomycin.

### Cytometry

Cells were detached 48 h p.tr from wells in PBE buffer (PBS + 1% FCS, 1 g/l NaN_3_, 2 mM EDTA), pelleted at 300 *g* for 3 min, washed in PBE, incubated with the indicated fluorochrome-labeled antibodies (3 μg/ml 2F5 or 4E10, 5 μg/ml anti-HA-MAb 3F10) for 1 h at room temperature and washed 2 times with cold PBE. 2F5 and 4E10 antibodies were labeled with AlexaFluor-647 (Protein Labeling Kit, Invitrogen) according to the manufacturer’s instructions. A mean of 6 fluorochromes was attached to each IgG molecule, as measured by photometric analysis (NanoDrop software v3.7.1). anti-HA-antibody was purchased as conjugate with Fluorescein (Roche). Apoptosis staining was done by the use of 7-AAD and Annexin-V-APC antibody (both diluted 1:100, both BD Biosciences). Finally, cells were subjected to cytometric analysis in FACS Canto II (BD, with FACS Diva software v6.0).

### Sucrose gradients

After purification through a sucrose cushion, VLPs were loaded onto a 10 ml 10-50% sucrose gradient and ultra-centrifuged at 100,000 *g* for 2.5 h. 20 fractions of 0.5 ml each were collected. For Western Blot analysis, particles were pelleted by trichloroacetic acid precipitation. A quarter of volume of 100% (w/v) TCA was added to the samples, incubated for 10 min at 4°C and spun at 14,000 *g* and 4°C for 5 min. The pellet was washed two times with cold acetone, dried at 95°C for 5 min and resuspended in SDS sample buffer. For EM experiments, fractions 13–17 were pooled, diluted in PBS ad 12 ml, ultra-centrifuged at 100,000 *g* for 1.5 h and resuspended in PBS.

### Electron microscopy

Sucrose-cushion purified VLPs were incubated with primary antibody 4E10 for 1 h at 37°C and further purified on a sucrose gradient. Pooled and resuspended VLPs were adsorbed to grids after fixation with 2% glutaraldehyde. Grids were washed in TBS (Leica EM IGL), blocked with 3% gelatine in TBS for 1 h at RT, and incubated with an anti-human IgG immuno-gold conjugate (particle size: 10 nm; Aurion, Wageningen, Netherlands) for 1 h at room temperature. After three washes in TBS, grids were contrasted with phosphoric tungstic acid and examined in an electron microscope (Zeiss EM 10C/CR).

### VLP production

VLPs for immunization purposes were produced in 293 F cells with the use of a codon-optimized, Rev-independent gene for Gag(IIIB) [24]. For pseudotyped VLPs, plasmids encoding for Gag and Env were mixed in a ratio of 2:1 in a co-transfection assay. VLPs were harvested 72 h p.tr., cleared by centrifugation at 3,000 *g* for 15 min, loaded onto a 30% sucrose in PBS cushion (5 ml for 30 ml of supernatant) and ultra-centrifuged at 100,000 *g* for 2 h. The pellet was resuspended overnight in PBS and stored at −80°C, with an aliquot analyzed by SDS gel electrophoresis and Coomassie staining for quality control. Both VLPs and DNA for immunization purposes were checked for low endotoxin levels with aid of Limulus Amebocyte Lysate QCL-1000 assay (Lonza Group Ltd, Basel, Switzerland), with a result of <20 EU/ml.

### Rabbit immunizations

The animal study was carried out in strict accordance with the UK Animals (Scientific Procedure) Act 1986, and the protocol was approved by the local Ethical and Welfare Committee of the University of Cambridge and the UK Home Office (Project license no. 80/2238). All efforts were made to minimize suffering. New Zealand rabbits (Harlan UK Ltd, Belton, Leicestershire, UK) were kept under pathogen-free conditions and pre-bled by ear vein lancing at the age of 12 weeks. Animals were grouped into 4 groups of 6 animals each and immunized at weeks 0 and 4 with 500 μg of plasmid DNA by i.m. saline injection in *quadriceps* muscles, followed by i.m. booster immunizations at weeks 12 and 16 with 100 μg of VLPs (Gag protein, analyzed in Coomassie gel), adjuvanted with 1% Carbopol 974 adjuvant. The integrity of pseudotyped VLPs in Carbopol was checked by sucrose gradient ultracentrifugation and following Western Blot analysis. Rabbits were terminally bled by cardiac puncture under isoflurane anesthesia and then euthanized. Serum was obtained by incubation of blood for 1 h at 37°C, 1 h on ice, centrifugation at 20,000 *g* for 15 min, and complement inactivation by incubation of supernatant at 56°C for 30 min. Unfortunately 2 animals from group 2 and 3 and one animal from group 1 had to be euthanized prior to finalization of the immunization protocol for reasons not related to the study and were therefore not included in data analysis. Statistical analysis was performed based on the correct number of animals in each group.

### Removal of anti-cell antibodies

HEK293F cells were stacked by centrifugation at 300 *g* for 3 min, supernatant was aspirated and cells were stored at 4°C for up to 5 days until usage. Stacked cells were dissolved in an equal volume of animal serum and shaken 3 times for 4 h at 800 rpm at room temperature, until the signal of anti-cell antibodies was below two times the background signal in cytometric analysis.

### Neutralization assays

Virus neutralization assays were performed in the laboratory of D.C. Montefiori (Duke University School of Medicine, Durham, USA), according to the published protocol [25]. Assays were done with Env-pseudotyped viruses in TZM-bl cells for isolate MW965.26 and with Env.IMC.LucR viruses in A3R5 cells (CEM human lymphoblastoid cell origin) for isolate SF162. Dilutions mediating inhibition of infection by 50% (ID50) were subtracted by ID50 of pre-bleed sera or in any case subtracted by a minimum of 20 (detection limit).

### Statistical analysis

Significance of anti-gp41 responses in ELISA and neutralizing activity in neutralization assays was determined using the Wilcoxon Two Sample Test. Each group was compared to its respective pre-bleeds to assess a potential neutralizing activity, and all groups were compared to each other to detect inter-group differences in neutralizing activity.

## Results

### Design of gp41 constructs with enhanced MPER exposure

**Figure 1:**
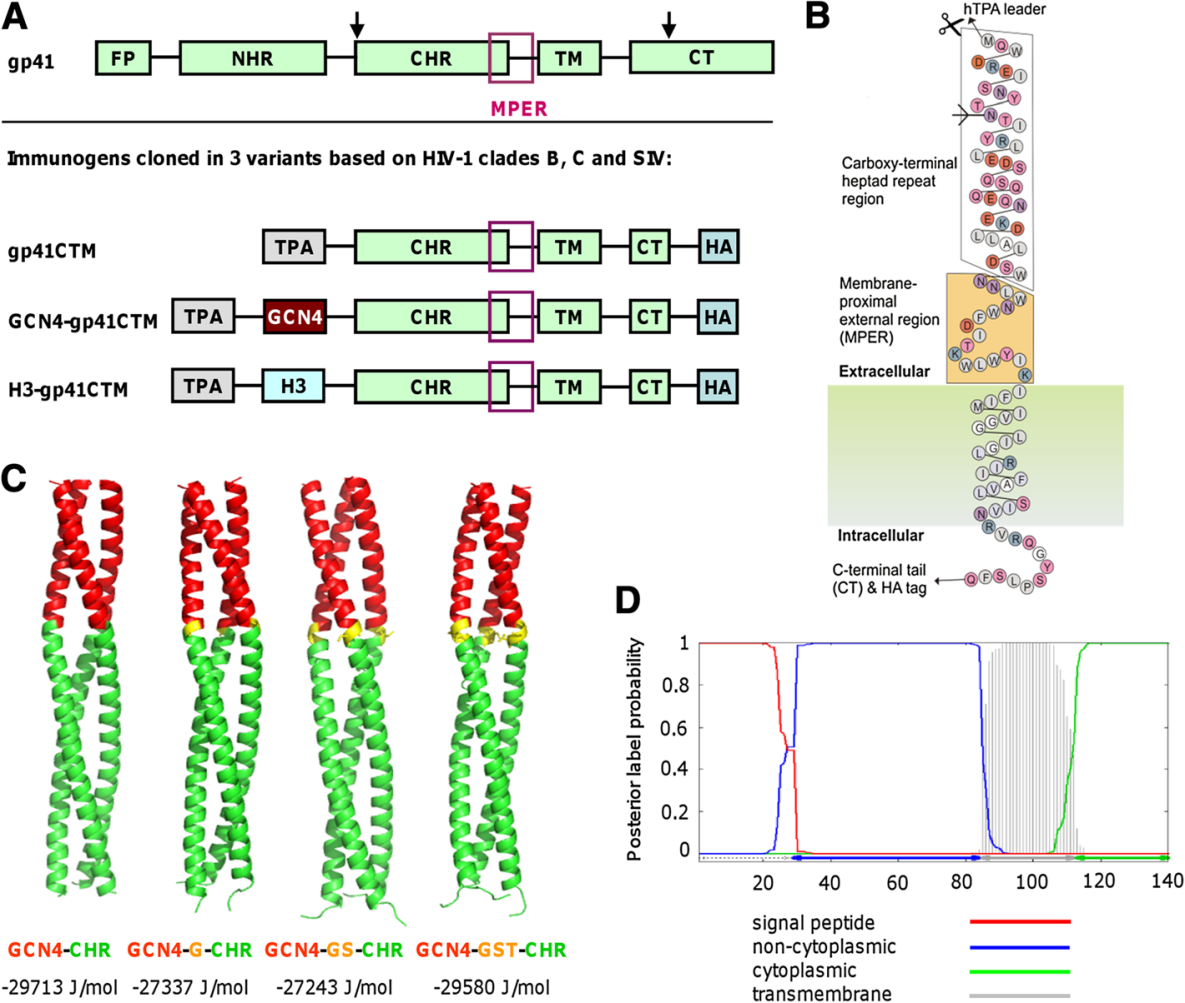
**Design of gp41-derived immunogens. (A)** Three genes based on gp41 were constructed in this study containing the CHR and TM regions (“gp41CTM”). Resulting proteins are directed by a TPA signal peptide towards the cell membrane and can be detected by a C-terminal HA tag. Two of these are stabilized in a trimeric conformation by an N-terminal heterologous zipper motif (GCN4- or H3-derived). **(B)** Schematic representation of truncated gp41 proteins on the surface of cellular membranes. The amino acid sequence is derived from isolate 96ZM651 (clade C). **(C)** The optimal fusion site of the GCN4 zipper domain and the gp41 CHR part was modeled by comparative modeling with the existing structure GCN4-NHR, and free energy was calculated with aid of DOPE score. **(D)** Correct cleavage of heterologous signal peptide, protein topology and membrane incorporation were predicted for the gp41CTM (based on 96ZM651) protein (data shown) and zipper-stabilized derivatives (data not shown).

Based on published gp41 constructs (33), novel gp41 immunogens were designed based on the sequence of three molecular clones: 96ZM651 (HIV-1 clade C) was selected for antigen development, because anti-MPER bnAbs have been originally isolated in clade C-infected patients [26] and clade C strains represent the majority of global HIV infections. 89.6 (HIV-1 clade B) and SIVmac239 (“SIV”) derived antigens served as controls. A signal peptide from the human tissue plasminogen activator gene (TPA) was N-terminally fused to the gp41 moiety to ensure that the protein is efficiently directed towards the cell membrane in mammalian cells. A hemagglutinin (HA) tag of nine amino acids was added to the protein’s C-terminus for uniform detection of the corresponding proteins (Figure 1). As described earlier, the C-terminal tail partially loops back to the extracellular and extraviral space, enabling access to antibodies [27]. As it is unknown whether the CHRs adopt a trimeric or monomeric conformation in the pre-fusion state, we designed trimer-stabilized variants in addition to the non-stabilized structure. Trimerization is generally thought to be beneficial for proper display of immunologically relevant sites within both gp41 and gp120, which - at the same time - assists the occlusion of irrelevant sites believed to distract B cells from vulnerable sites. Two heterologous trimerization domains were introduced between the signal peptide and the gp41-derived part: GCN4 and H3 zipper domains have been used previously to stabilize a gp41 post-fusion [19] or intermediate structure [13,28], respectively. For the H3 zipper domain, the orientation was adopted from an existing fusion protein by Hinz et al. [13], including a three amino acid spacer. Concerning the GCN4 domain, instead of fusing it to the NHR building the inner core of 6HB [6], it was fused N-terminally to the CHR, anticipating the stabilization of an exposed pre-hairpin conformation [28]. The correct fusion site was deduced by combining existing crystal structures of both the GCN4 domain and gp41. A molecular model of the ectodomain of a GCN4-gp41CTM fusion protein (comprising the GCN4 zipper domain and residues 1–50 of gp41 CHR) was built based on a solved structure of a GCN4-NHR fusion protein (1ENV, residues 1 to 80, [19]). Another three models were built with insertion of one, two, or three flexible aa (G, S, T) as linkers using the Modeller server, and the minimized energy was calculated by the aid of DOPE score (Figure 1C). The model without insertions showed the minimal energy and was chosen for cloning as it likely represents the structure with the highest stability. Its calculated energy (−29.7 kJ/mol) was close to that calculated for the modeling template GCN4-NHR of −30.8 kJ/mol. Two further crucial steps, the cleavage of the signal peptide and the membrane orientation, were controlled with appropriate prediction software. Proper signal peptide cleavage was predicted for all constructs. However, a wrong membrane orientation was predicted for the SIVmac239-based gp41CTM construct. For this reason, the cytoplasmic tail was elongated for 9 aa compared to 96ZM651- and 89.6-based templates. Correct membrane topology was predicted for all constructs after this modification (Figure 1D exemplarily for basal construct gp41CTM based on isolate 96ZM651).

### Membrane exposure and selective recognition by bnMAbs

**Figure 2:**
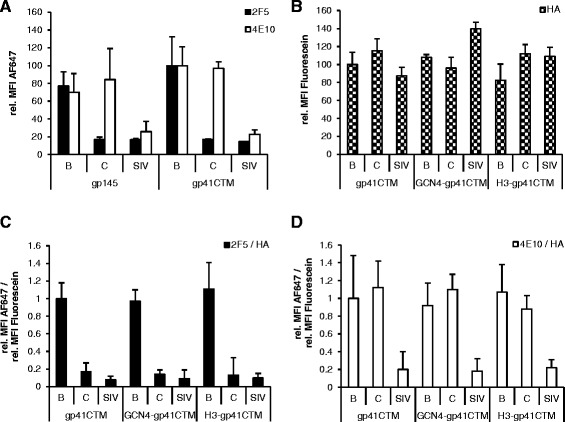
**Membrane exposure of novel constructs and recognition by bnMAbs.** 293 T cells were transfected with equal masses of pcDNA3.1(+) expression plasmids containing envelope genes and stained 48 h p.tr. with AlexaFluor647-labelled 2F5 or 4E10 bnMAbs and Fluorescein-labeled anti-HA antibody. **(A)** Comparison gp41CTM to gp145. MFI for all constructs was normalized to those of gp41CTM(89.6). **(B)** Detection of gp41 derivatives of clades B (89.6), **C** (96ZM651) and SIVmac239 by extracellular staining with anti-HA antibody, normalized to gp41CTM(89.6). **(C, D)** 2F5 and 4E10 binding to gp41 variants, normalized to the fluorescence intensity of their simultaneous anti-HA staining. Error bars indicate the standard deviation of three independent experiments.

Expression and membrane location of gp41 variants is a prerequisite for incorporation into VLPs, display of relevant epitopes and usability in immunization studies. In a first experiment, *in vivo* properties of gp41CTM were evaluated in comparison to gp145, which is a codon-optimized version of gp160 with mutated cleavage site and truncated C-terminal tail which increases surface presentation [29]. The clade C molecular clone 96ZM651 was intended to serve as source for cloning the Env/gp41 derivatives. The clade B isolate 89.6 and SIVmac239 were chosen as additional controls for proper display, as they possessed either both or none of the epitopes for 2F5 and 4E10, respectively. HEK293T cells were transfected with expression plasmids encoding gp145 and gp41CTM and stained with the mentioned bnMAbs. gp41CTM exhibited a slightly, yet non-significantly, enhanced mean fluorescence intensity (MFI) in cytometric analysis compared to gp145 (Figure 2A). The staining with 2F5 and 4E10 showed a selective binding of 2F5 to 89.6-based constructs only, whereas 4E10 as expected bound to both 89.6 and 96ZM651-based constructs. Following staining of the HA-tag with an anti-HA-antibody, all variants showed proper membrane localization and similar expression levels (Figure 2B). For linking bnMAb binding with the level of Env expression, MFIs of 2F5 and 4E10 were normalized to the anti-HA intensity. The SIVmac239-based variants bound to none of the tested bnMAbs and were only detectable by the HA-tag, as expected. Signal intensities were similar for the basal and trimer-stabilized constructs, which indicated a similar accessibility of bnMAb epitopes in these constructs, irrespective of trimerization domains.

### Incorporation of oligomeric gp41 derivatives into VLPs

**Figure 3:**
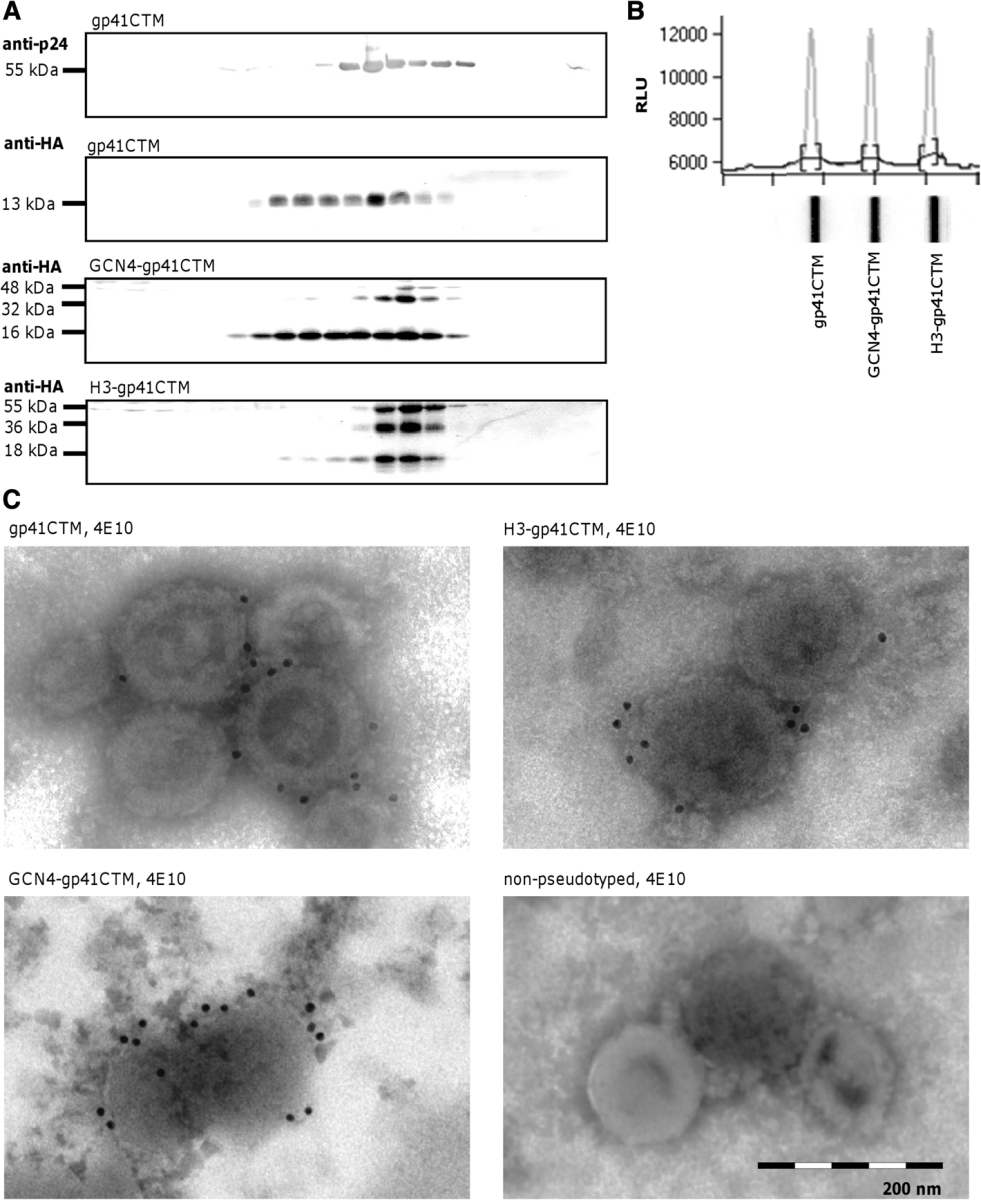
**Biochemical characterization of VLPs containing gp41 variants. (A)** Incorporation of gp41 derivatives into VLPs. VLPs were produced as described above and analyzed in 10 to 50% sucrose gradients. 20 fractions were collected and loaded on a reducing SDS gel, respectively. gp41 variants were detected by anti-HA antibody and Pr55Gag by anti-p24 MAb 13/5 in subsequent Western Blot analysis. Co-banding of Gag (only shown for basal construct gp41CTM) and gp41 variants in fractions 11–15 indicates incorporation into virus-like particles. Higher molecular weight bands for GCN4- and H3-gp41CTM indicate stable dimeric and trimeric conformations. VLPs produced by transfection of Gag without gp41 were negative for anti-HA-antibody staining (data not shown). **(B)** Quantification of gp41 derivatives in VLPs. 0.25 μg of indicated lysed pseudotyped VLPs were loaded onto a slot blot, and stained with 4E10 and anti-human-HRP antibody. **(C)** Functional preservation of bnMAb epitopes on VLPs. VLP morphology and epitope preservation were verified by immuno-gold labeling and electron microscopy. VLPs were purified and incubated with human bnMAb 4E10 and gold-labelled anti-human antibody. VLPs pseudotyped with gp41CTM, GCN4-gp41CTM and H3-gp41CTM showed specific staining with 4E10. VLPs without pseudotyping exhibited no labeling (right).

Further analysis of gp41 variants was restricted to the constructs based on the molecular clone 96ZM651, as these were chosen for following immunization studies. As it is known that HIV-1-derived VLPs band at a density of 1.14-1.18 g/cm^3^ corresponding to ~35% glucose in PBS [30], the incorporation of novel gp41 immunogens into VLPs was addressed by sucrose gradients and Western Blot analysis. VLPs were produced by co-transfection of HEK293T cells with plasmids encoding Gag and gp41CTM by RNA- and codon-optimized genes, respectively. Gag as well as gp41 derivatives that were incorporated into VLPs prior to or during budding were detected in Western Blot analysis using polypeptide specific antibodies. All variants showed a co-banding of both proteins in fractions 12–14, suggesting successful incorporation into VLPs (Figure 3A). Higher molecular weight bands for GCN4-gp41CTM and H3-gp41CTM indicated the formation of stable dimers and trimers. For a relative quantification of incorporated gp41 proteins, VLPs were purified, normalized to Gag content, lysed and loaded onto a slot blot, followed by detection with 4E10 (Figure 3B). All gp41 variants exhibited similar signal intensities and are therefore supposed to be incorporated at similar amounts into VLPs. Since the recognition by 4E10 of a similar soluble standard protein (GCN4-gp41CTM-FD) was substantially lower than that of gp41CTM and its derivatives (data not shown), it was not possible to determine the absolute number of gp41CTM molecules on the surface of VLPs. The morphology of VLPs pseudotyped with gp41 derivatives and the antigenic properties of incorporated proteins were further evaluated by electron microscopy. VLPs were labeled with 4E10 and secondary immuno-gold antibody. gp41 derivatives had no apparent impact on particle morphology and size of 100–150 nm in diameter (Figure 3C). Staining with 4E10 showed specific labeling of VLPs pseudotyped with all three types of gp41 derivatives, whereas non-pseudotyped VLPs were not labeled.

### Induction of anti-gp41 antibodies

**Figure 4:**
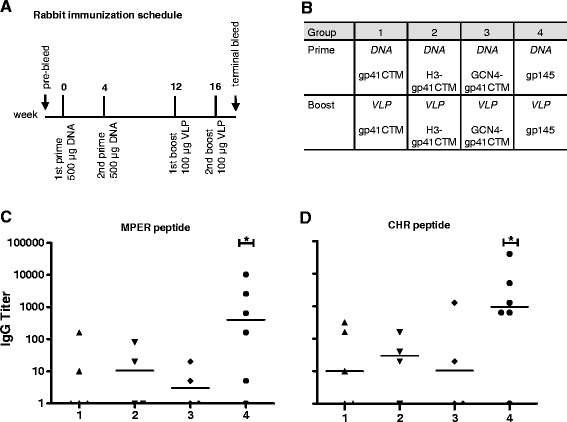
**Anti-gp41 antibodies induced by immunization. (A)** Immunization protocol. Rabbits were immunized twice with DNA encoding for trimer-stabilized constructs and twice with homologous VLPs at indicated time points. **(B)** Three groups receiving gp41CTM and trimer-stabilized constructs were assigned. One group receiving gp145 DNA and VLPs served as control group. **(C,D)** Terminal bleeds of rabbits were analyzed by IgG ELISA. Peptides spanning the MPER **(C)** and CHR **(D)** served as coating peptides. Anti-rabbit-HRP coupled antibody was used as secondary antibody. Titers are calculated as reciprocal dilutions of specific signals which are at least two-fold above pre-bleed signals. Asterisks above single groups mark a mean titer significantly above background level (p < 0.05).

The immunogens were evaluated for immunogenicity in a rabbit immunization study. A DNA prime and VLP boost regimen was chosen, because heterologous immunization schedules using other Env constructs had induced higher antibody titers compared to DNA or VLPs alone [31]. After attesting low endotoxin levels in the immunogens formulation, VLPs were formulated with 1% 974 carbopol as adjuvant. Carbopol was chosen because of its optimal formulation properties with lipid membranes, compared to Freund’s adjuvant or oil in water formulations. The integrity of VLPs and incorporated proteins after mixing with Carbopol was verified by sucrose gradient and Western Blot analysis (data not shown). Rabbits were first primed twice with 500 μg of DNA at weeks 0 and 4, then boosted two times with 100 μg of VLPs formulated in Carbopol, at weeks 12 and 16 (Figure 4A). Bleeds were taken 2 weeks before the first immunization and 2 weeks after the last immunization. The induction of anti-gp41 Abs is a prerequisite for effective neutralization, and was analyzed by IgG ELISA. Reactivity of sera was dissected using an MPER (Figure 4C) and a CHR peptide immobilized to the solid phase (Figure 4D). Whereas animals that received gp145 (positive control) developed significant titers against both peptides, only low titers were obtained for the groups that were immunized with gp41 derivatives. Immune responses in non-reacting animals might have been too low to be detected in unfractionated sera.

### Neutralizing capacity of unfractionated immune sera

**Figure 5:**
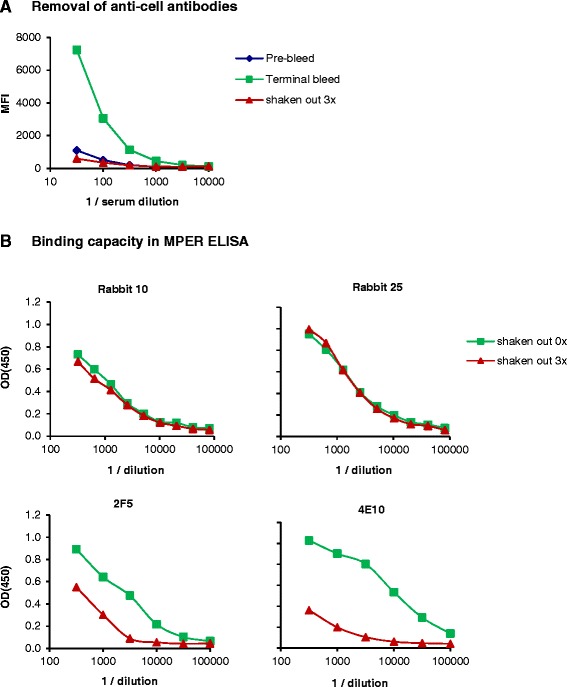
**Removal of anti-cell antibodies. (A)** Effective removal of anti-cell antibodies in sera of immunized animals. Sera of rabbits were incubated with human 293 cells for 4 h at room temperature with moderate shaking. Three rounds of incubation were sufficient to remove all significant anti-cell activity, measured by staining of human 293 T cells with dilutions of sera and PE-labeled anti-rabbit antibody. Mean fluorescence intensity (MFI) of a representative serum is shown. **(B)** Anti-MPER antibodies 2F5 and 4E10, but not anti-gp41 Abs induced by immunization were depleted by incubation with cells. Serial dilutions of 2F5, 4E10 and immune sera in PBS were incubated three times with an equal volume of HEK293F cells. Supernatants were read out in a gp41 ELISA with MPER peptide as coating reagent, and anti-human-HRP coupled antibody as secondary antibody.

**Figure 6:**
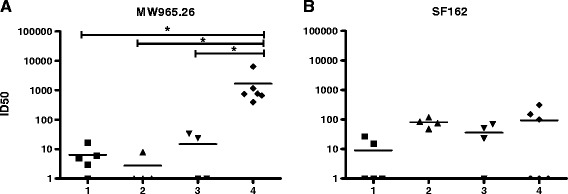
**Induction of low neutralizing antibody titers in rabbit sera after immunization with gp41 derivatives.** After removal of anti-cell antibodies, sera were tested in neutralization assays with two viral strains. Dilutions mediating inhibition of 50% infection (ID50) subtracted by ID50 of pre-bleed sera are shown. Sera of all groups were tested against tier-1 isolates **(A)** MW965.26 (clade C) and **(B)** SF162 (clade B). Asterisks mark significant difference between groups (p < 0.05).

Immunization of rabbits with VLPs derived from heterologous production systems (HEK293T cells) can induce antibodies directed against HEK293T cell derived membrane proteins, and these antibodies interfere with neutralization assays. As anti-cell antibodies were detected in some immunized animals (Figure 5A), sera were incubated with HEK293T cells three times to absorb and deplete anti-cell antibodies [32]. Antibodies directed against a 30-mer MPER-peptide, that were present in animal sera, were not detectably affected by this procedure (Figure 5B, upper panel), while anti-cell antibodies were successfully removed (Figure 5A). As a control, 2F5 and 4E10 recombinant antibodies were incubated with HEK293 cells as well, and were depleted partially in this assay. 4E10 was depleted more pronouncedly than 2F5, as 4E10 has a higher membrane-binding activity than 2F5 (Figure 5B, lower panel). Subsequently, all sera were tested in standardized assays for neutralizing activity in a TZM-bl assay. A tier-1 molecular clone of clade C (MW965.26) and a tier-1 clone of clade B (SF162) were chosen for readout. Clade C isolate MW965.26 contains the 4E10 epitope but not the 2F5 epitope, like 96ZM651, whereas clade B isolate SF162 contains the epitopes for both 4E10 and 2F5, like isolate 89.6 [33]. In general, results from neutralization tests tended to reflect titers of anti-MPER-peptide antibodies as measured in ELISA. Effective neutralization was observed only for the rabbit group receiving gp145 DNA and homologous VLPs (VLP-gp145), with two rabbits showing neutralization at a 50% inhibitory dilution (ID50) of >1,000 against MW965.26 (Figure 6A). For all three rabbit groups receiving gp41-derived DNA prime and VLP boost, neutralizing activities were weak. However, statistically significant neutralization at low level was observed for group 1 against MW965.26 and group 2 against SF162. An ID50 of up to ~100 could be detected in several further animals against both isolates. Interestingly, neutralizing activity of sera of group 1–3 after gp41 immunization appeared to be more pronounced against the clade B isolate than against the clade C isolate, indicating better cross-clade neutralizing activity than obtained with group 4 after gp145 immunization.

## Discussion and conclusions

Previous attempts to elicit nAbs against the 2F5 [34,35] and the 4E10 epitope [36,37] have largely failed, emphasizing the challenges associated with generating a focused response to MPER-neutralizing epitopes. However, an approach that combines the exposure of neutralizing sites on the immunogen fixed in a favorable structure following truncation of unwanted or immunodominant epitopes and presented in a membrane environment may be effective. Therefore, this work focused on the creation and immunological evaluation of novel gp41-derived immunogens, which meet the above mentioned requirements of such an MPER-based immunogen.

Various steps were undertaken to improve exposure of conserved regions, to stabilize relevant conformations and to focus B cell responses: (i) gp41 was truncated N-terminally to eliminate dispensable sites; (ii) the CHR was included to promote proper trimerization and MPER folding and (iii) the gp41 C-terminus was truncated to allow for efficient incorporation into VLPs [29] and to support an optimal configuration for 4E10 epitope exposure [38].

Cytometric analyses validated membrane topology and binding to bnMAbs 2F5 and 4E10. Signals for 2F5 and 4E10 epitopes as well as the C-terminally fused HA tag were low, but significant and specific, indicating a low level of presentation, yet proper membrane incorporation. The recognition of the HA-tag located in the antigenic region of the “Kennedy sequence” in the cytoplasmic tail supports the hypothesis of additional membrane-spanning domains leading to exposure of the tag [39–42]. Electron microscopy experiments of VLPs stained with 4E10 clearly demonstrated the gp41 derivatives to be presented on the surface of VLPs. Higher molecular weight bands of trimer-stabilized envelopes (Figure 3) upon assessment of VLPs by Western Blot analysis indicated a high stability of dimeric and trimeric assemblies. According to previous finding by Lenz et al. [15], the observed shift towards di- and trimeric conformations following N-terminal fusion of the zipper domains (gp41CTM) may be a result of GCN induced trimer formation, trimer stability or both. Generally, trimeric conformations are considered superior to monomeric gp41 derivatives as (i) they present and stabilize discontinuous epitopes and (ii) occlude immunodominant epitopes disclosed by the monomeric protein [43]. Taken together, the *in silico* models of designed gp41 variants could be implemented into proteins with the desired biochemical specifications.

For immunogenicity studies a DNA prime and VLP boost regimen of rabbits was chosen as a positive effect of DNA priming on antibody titers had been observed by others studying immunogenicity of MPER-containing proteins [31]. Inter-individual variations in gp41 specific IgG responses within groups were notable, which had also been observed before [43]. gp41 specific antibody titers induced by any gp41 DNA prime/VLP boost combination were generally weak compared to titers determined in the gp145 control group. As reactivities against MPER and CHR were similarly low in groups receiving gp41CTM and trimer-stabilized derivatives, the low reactivity may be attributed to low protein expression and exposition on the VLP surface. This limitation may be overcome by using heterologous transmembrane domains from e.g. MMV or Influenza virus, which have been previously shown to mediate enhanced incorporation and display of truncated Env proteins [29]. Moderate neutralization found in animals immunized with VLPs presenting gp145 on their surface may benefit from (i) increased numbers of presented Envelopes and (ii) from the additional epitopes such as e.g. V3 and others presented by the gp120 moiety of gp145.

Sera were pre-incubated several times with non-transduced HEK293T production cells to remove anti-cell antibodies from immune sera prior to neutralization assays. Figure 5a demonstrates as exemplified by sera obtained from rabbit 10 that the pre-incubation procedure leads to a significant reduction of anti-cell antibodies below the level detected in the corresponding prebleed serum. Whereas Figure 5b (lower panel) demonstrates for 2 MPER specific MAbs, 2F5 and 4E10, that the pre-incubation procedure reduces the binding of these MAbs to a 30-mer MPER-peptide, the upper panel depicting the reactivity of selected antisera before and after the pre-incubation procedure does not provide any evidence for removal of MPER-specific antibodies. However, despite these ELISA binding data we cannot completely rule out the possibility that there was a subspecies of 2F5- or 4E10-like antibodies, which was not captured by the 30-mer linear MPER-peptide but may have been depleted by pre-incubation procedure. As a consequence, future immunization studies engrafting the MPER on VLPs should emphasize the production of VLPs in cell-lines derived from the homologous species to be immunized.

Neutralization in our study was observed only in sera which proved to contain ELISA reactive antibodies. However, whereas a previous study claimed that trimer stabilization of VLP-exposed MPER peptides exhibit a beneficial effect on the formation of neutralizing antibody titers [31], our constructs induced comparable neutralizing titers irrespective of the absence or presence of the fused trimerization domain (Figure 4). As the gp41CTM core construct used in this study comprised a more extended CHR portion (55 residues) compared to the more truncated forms (42 and 24 residues) used by others [31], the longer CHR per se may already be sufficient to support or stabilize gp41CTM trimerization and proper display *in vivo*.

Recent studies underscored the importance of the membrane environment to properly present MPER immunogens to B cells. Consistently it was demonstrated that these approaches can induce a low titer of neutralizing antibodies, yet highlight the difficulty of achieving high titers [44,45]. Some other immunogen designs where the MPER had been fused to various heterologous carriers elicited strong peptide specific reactivities, yet had not been successful in showing HIV-1 neutralizing activity [46,47] The gp41 constructs presented here were antigenic for 4E10 and induced a basic level of neutralization, which needs to be further enhanced - a common phenomenon among recent gp41 immunization studies [48–52]. Finally, a study by Bomsel et al. showed that not only neutralization, but also transcytosis-blocking properties and antibody-dependent cellular-cytotoxicity may protect from SHIV infection after animals had been immunized with virosomes containing an MPER peptide [53]. Thus*,* assessment of IgG neutralizing activity of sera *in vitro* may not reflect protection at mucosal sites *in situ*. The evaluation of functional parameters other than neutralization, such as ADCC, ADVI and transcytosis blocking, may be of value in future MPER targeting studies.

## Additional file

Additional file 1: Table S1.
**Overview on synthetic oligonucleotides used for cloning.**
