# Supplementary material for: Development and immunological assessment of VLP-based immunogens exposing the membrane-proximal region of the HIV-1 gp41 protein
Source: J Biomed Sci. 2014 Aug 27;21(1):79. doi: 10.1186/s12929-014-0079-x (PMC4256929; doi:10.1186/s12929-014-0079-x)
Supplement: Additional file 1: Table S1. — Overview on synthetic oligonucleotides used for cloning. [file 12929_2014_79_MOESM1_ESM.pdf]

Supplementary table 1. Overview on synthetic oligonucleotides used for cloning.

ID	Sequence	Name
3E8	CTGGCTAGCGCCGCCACCATGGATGCCATGAAGCGGGG	NheI-Kozak-TPA fw
2H4	GGTACCTTACTAGGCGTAGTCGGGCACGTCGTAGGGGTAGCTGG CGGGCAGCAGGGTCTGGAAGC	KpnI-gp41CTM(89.6)-HA rev
2H5	GGTACCTTATTAGGCGTAGTCGGGCACGTCGTAGGGGTATGGGTT CGGGATAAGGGTCTGAAACGTGAGTATCC	KpnI-gp41CTM(96ZM)-HA rev
2H6	GGTACCCTATCAGGCGTAGTCGGGCACGTCGTAGGGGTAGTCCT GTTGGATATGGGTCTGCTGGAAATAAGAGG	KpnI-gp41CTM(SIVmac239)-HA rev
3B6	TCGTTCTCGATGTGGTAGATCTTGCTCAGGATCTCCTCGATCTTGT CCTCGATCTGCCTGGCGCCCCTTCTGAACCGGGCGT	TPA-GCN4-Fusion rev
3B7	CCTGAGCAAGATCTACCACATCGAGAACGAGATCGCCCGCATCAA GAAGCTGATCATGGAATGGGAGCGGGAGATCGACAAC	GCN4-gp41CTM(89.6)-Fusion fw
3B8	CCTGAGCAAGATCTACCACATCGAGAACGAGATCGCCCGCATCAA GAAGCTGATCATGCAGTGGGACCGGGAGATCAGCAAC	GCN4-gp41CTM(96ZM)- Fusion fw
3B9	CCTGAGCAAGATCTACCACATCGAGAACGAGATCGCCCGCATCAA GAAGCTGATCCAGGAATGGGAGCGGAAGGTCGACTTCC	GCN4-gp41CTM (SIVmac239)-Fusion fw
3H4	CTCGCTGAACTCCTTCTCGATCTGGTGGAACTTCTCGTTGGTCTTC TCGATCACGCGGTTCAGCTTGCCGTTGATCTGGTCGATCCGGGCT CCCCGTCTGAACCGGGCGT	TPA-H3-Fusion rev
3H5	TTCCACCAGATCGAGAAGGAGTTCAGCGAGGTGGAGGGCCGCAT CCAGGACCTGGAGAAGTACGTGGAGGACACCGGCAGCACCATGG AATGGGAGCGGGAGATCGACAAC	H3-gp41CTM(89.6)-Fusion fw
3H6	TTCCACCAGATCGAGAAGGAGTTCAGCGAGGTGGAGGGCCGCAT CCAGGACCTGGAGAAGTACGTGGAGGACACCGGCAGCACCATGC AGTGGGACCGGGAGATCAGCAAC	H3-gp41CTM(96ZM)-Fusion fw
3H7	TTCCACCAGATCGAGAAGGAGTTCAGCGAGGTGGAGGGCCGCAT CCAGGACCTGGAGAAGTACGTGGAGGACACCGGCAGCACCCAGG AATGGGAGCGGAAGGTCGACTTC	H3-gp41CTM(SIVmac239)-Fusion fw
